# Molecular docking analysis of Bcl-2 with phyto-compounds

**DOI:** 10.6026/97320630016468

**Published:** 2020-06-30

**Authors:** Chandrasekaran Kirubhanand, Jayaraman Selvaraj, Umapathy Vidhya Rekha, Veeraraghavan Vishnupriya, Venkatachalam Sivabalan, Mathayan Manikannan, Devarajan Nalini, Periyasamy Vijayalakshmi, Manikkam Rajalakshmi, Rajagopal Ponnulakshmi

**Affiliations:** 1 Department of Anatomy, All India Institute of Medical Sciences, Nagpur, India; 2 Department of Biochemistry, Saveetha Dental College and Hospitals, Saveetha Institute of Medical and Technical Sciences, Saveetha University, Chennai - 600 077, India; 3 Department of Public Health Dentistry, Sree Balaji Dental College and Hospital, Pallikaranai, Chennai-600 100, India; 4 Department of Biochemistry, KSR Institute of Dental Science and Research, Tiruchengode-637215. Tamil Nadu, India; 5 Centre for Drug Discovery and Development, Sathyabama Institute of Science and Technology, Chennai-600 119, India; 6 Central Research Laboratory, Meenakshmi Ammal Dental College, Meenakshi Academy of Higher Education and Research (Deemed to be University), Chennai-600 095, India; 7 DBT-BIF Centre,PG and Research Department of Biotechnology and Bioinformatics, Holy Cross College (Autonomous), Trichy, Tamil Nadu, India; 8 Central Research Laboratory, Meenakshi Academy of Higher Education and Research (Deemed to be University), West K. K. Nagar, Chennai-600 078, India

**Keywords:** Breast cancer, bioactive compounds, Bcl-2, molecular docking

## Abstract

The Bcl-2 protein is liked in several cancers and drug resistance to therapy is also known in this context. There are many Bcl-2 inhibitors under clinical trials. It is of further interest to design new Bcl2 inhibitors from phyto compounds such as artesunate, bruceantin, maytansin, Salvicine, indicine N-oxide, kamebanin and oxyacanthine. We report the optimal binding features of these compounds with Bcl-2 for further consideration towards in vitro and in vivo validation.

## Background

Cancer related issues [1-6] are linked to the BCL-2 family of proteins [7- 12]. BCL-2 is a known cancer target with several potential inhibitors under validation. Therefore, it is of interest to design and develop new compounds from plant source with improved binding features with the BCL-2 protein. Hence, we report the molecular docking analysis of Bcl-2 with phyto-compounds.

## Materials and Methods:

### BCL-2 structure:

The structure data of Bcl-2 protein (PDB ID: 2W3L) was downloaded from the protein databank (PDB) [13] and processed for further analysis.

### Ligand data:

The structures of phytocompounds such as artesunate, bruceantin, maytansin, Salvicine, indicine N-oxide, kamebanin, and oxyacanthine were downloaded from the pubchem database in SDF format and converted to PDB format using the Online Smiles Translator.

### Active site prediction:

Binding site prediction of Bcl-2 was completed using MetaPocket 2.0 server [14].

### Molecular docking:

PatchDock [15,16] was used for the molecular docking analysis of BCL-2 with phytocompounds such as artesunate, bruceantin, maytansin, Salvicine, indicine N-oxide, kamebanin and oxyacanthine.

## Results and discussion:

**Figure 1:**
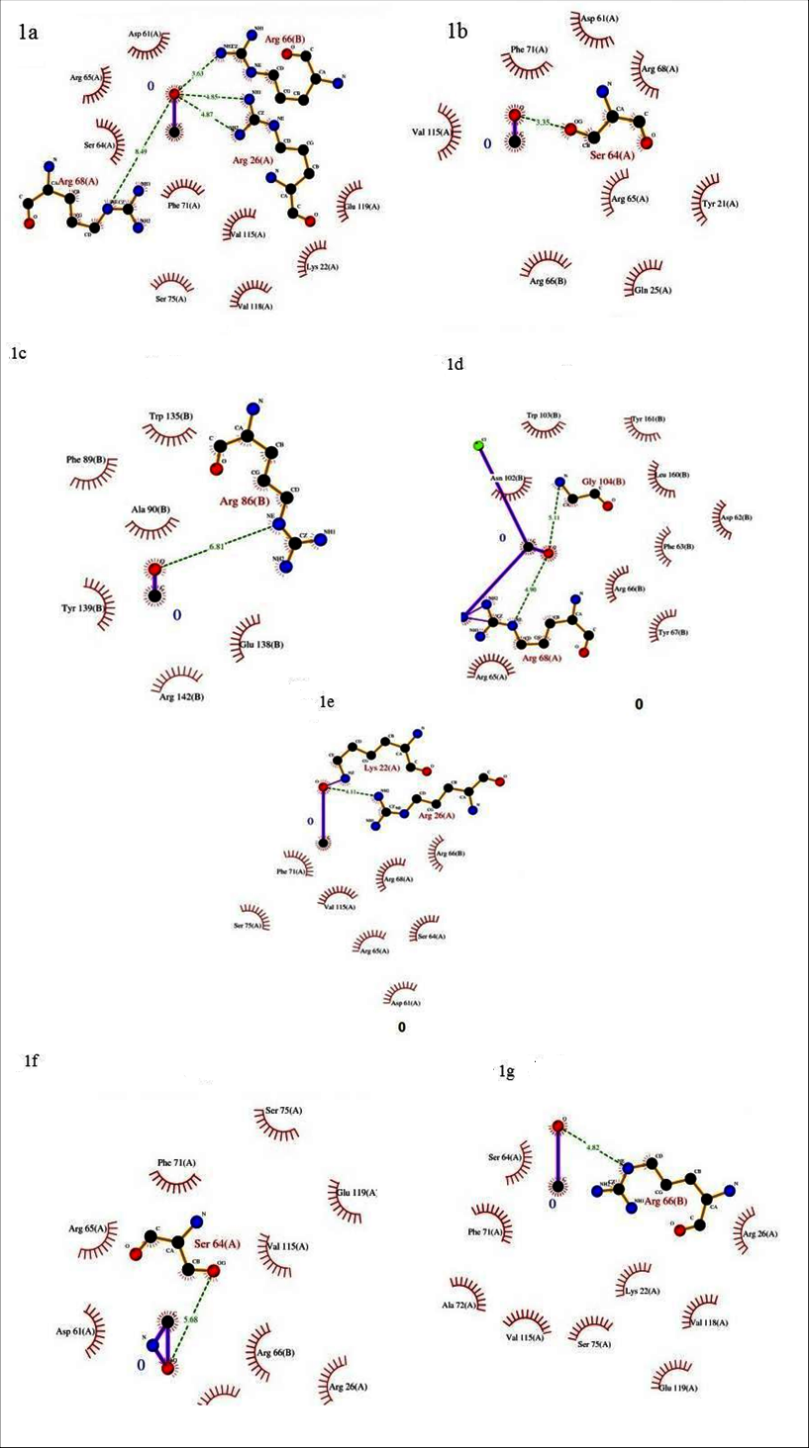
Interaction of Bcl-2 and (a) artesunate; (b) bruceantin; (c) maytansin; (d) salvicine; (e) indicine N-oxide; (f) kamebanin and (g) oxyacanthine is shown using ligplot.

BCL2 is a known cancer target. Therefore, it is of interest to design new Bcl2 inhibitors from phyto compounds such as artesunate, bruceantin, maytansin, Salvicine, indicine N-oxide, kamebanin and oxyacanthine. The predicted active site residues in BCL-2 are LYS 22, GLN 25, ARG 26, THR 55, ASP 62, SER 64, ARG 66, TYR 67, ARG 68, PHE 71, LEU 80, ARG 86, ASN 102, GLY 104, VAL 107 and GLU 111. The molecular docking analysis data of Bcl-2 with the phyto-compounds are given in Table 1. Detailed interaction between BCL-2 and the compounds are shown in Figure 1. The residues ARG 26, ARG 68, ARG 66, SER 64, ARG 86, ARG 68, GLY 104, LYS 22, SER 20, ASN 34, LYS 22, LYS 270, SER 351, ARG 469, and LYS 345 are found to be interacting with the phyto compounds (Figure 1). Thus, we report the optimal binding features of these compounds with Bcl-2 for further consideration towards in vitro and in vivo validation.

## Conclusions:

We report compounds with improved binding features with Bcl-2 for further consideration towards in vitro and in vivo analysis.

## Figures and Tables

**Table 1:** Molecular docking analysis of Bcl-2 with phyto-compounds

S. No	Compound Name	Score (kcal/mol)	ACE	Hydrogen bonds	Bond length	No of non-bonded interaction
1	Artesunate	4814	-211.33	ARG 26- NH-O	2.46	148
				ARG 68- NE-O	3.24	
				ARG 66- NH2-O	3.3	
2	Bruceantin	5714	-104.71	SER 64 OG-O	3.34	67
3	Maytansin	773.1	-110.4	ARG 86 NE-O	2.04	102
4	Salvicine	4946	-126.9	ARG 68 NE-O	2.17	131
				GLY 104 N-O	2.47	
5	Indicine N-oxide	4226	-130.62	LYS 22 NZ-O	1.75	37
				ARG 26 NH2-O	1.95	
6	Kamebanin	4002	-140.03	SER 64 OG-O	3.08	40
7	Oxyacanthine.	6368	-102	ARG 66 NE-O	2.79	31
